# New Hepatitis B Vaccine Schedule for Children with Celiac Disease

**DOI:** 10.5812/kowsar.1735143x.772

**Published:** 2011-10-01

**Authors:** ﻿Salvatore Leonardi, Michele Miraglia del Giudice, Mario La Rosa

**Affiliations:** 1 Department of Pediatrics, University of Catania, Catania, Italy; 2 Department of Pediatrics, University of Neaples, Neaples, Italy

**Keywords:** Hepatitis B Vaccines, Celiac Disease

Dear Editor,

The article by Ertekin et al. published in Hepatitis Monthly indicates that a new hepatitis B virus (HBV) vaccine schedule should be created for children with celiac disease (CD), and the response of all children with CD to the HBV vaccine should be routinely investigated [1]. The authors investigated a group of children with CD vaccinated against HBV and found that the anti-HBs titer was not detected in a significant percentage (38.5%) of children. In addition, the clinical presentation of the disease and dietary compliance of the patients seemed to play a role in nonresponsiveness to the HBV vaccine. Therefore, the authors suggest a different immunization schedule. Most studies indicate that major histocompatibility complex (MHC) and human leukocyte II antigens (HLA-II) are responsible for nonresponsiveness to HBV vaccine [2][3][4][5]. However, Nemes et al. [6] found that the percentage of seroconversion (95.5%) in a group of patients vaccinated after initiating dietary treatment was similar to that in healthy individuals, and overall, 36 out of 37 nonresponders consuming a controlled gluten-free diet showed seroconversion after administering a booster dose of vaccine. This finding shows that HLA DQ alleles do not have a primary role in nonresponsiveness to HBV vaccine. The nature of protection afforded by vaccination is complex and, although both humoral and cytotoxic T-cell responses play a role, long-term protection is dependent upon immunological memory. The general belief is that immunological memory persists for a long period after natural infection and administration of live vaccines. For many years, the response to HBV vaccination is determined by measuring the anti-HBs titer, and a patient with an anti-HBs level < 10 mIU/mL is considered to be a nonresponder. However, responsiveness to HBV vaccination is usually determined by testing within 2-6 months after receiving the third dose of the vaccine. Thus, the nonresponsiveness to HBV vaccine might be secondary to the tendency of older individuals having a weaker response to the vaccine or not developing immunity. In patients with anti-HBs level < 10 IU/L, a schedule for booster vaccinations should be proposed, but no consensus has been achieved on the management of nonresponders to date.

A high dose of HB vaccine is administered in healthy individuals, but this procedure is not uniformly successful [7]. Recently, 90% seroconversion was observed in a pilot study in patients after administration of a booster dose of vaccine by the intradermal (ID) route [8]. Large prospective randomized studies are required to validate this procedure to improve the efficacy and safety of the current standard protocol. The mechanism of induction of immunogenicity via intradermal administration is not clearly established but may be related to the sequestration of the antigens in the dermis for a long time after intradermal injection. The dermis contains large numbers of dendritic cells (the most potent antigen-presenting cells) that are important for the ID route of immunization, and these cells are thought to induce cell-mediated immune responses, particularly CD4+ and CD8+ T-cell responses. In addition, dendritic cells enhance antibody production by B cells through efficient induction of CD4+ T-cell modulation of B cells and thus a lower antigen dose is required to produce an immune response.
